# Impact of Adsorption of Straight Chain Alcohol Molecules on the Optical Properties of Calcite (10.4) Surface

**DOI:** 10.3390/nano12091460

**Published:** 2022-04-25

**Authors:** Junais Habeeb Mokkath

**Affiliations:** Quantum Nanophotonics Simulations Lab, Department of Physics, Kuwait College of Science and Technology, Doha Area, 7th Ring Road, Kuwait City P.O. Box 27235, Kuwait; j.mokath@kcst.edu.kw

**Keywords:** adsorption, calcite, DFT, TD-DFT

## Abstract

Calcium carbonate plays a central role in controlling the chemistry of the oceans, biomineralization and oil production, to name a few. In this work, using density functional theory with semiempirical dispersion corrections and simplified TD-DFT using Tamm–Dancoff approximation, we investigated the impact of the adsorption of straight chain alcohol (ethanol and pentanol) molecules on the optical properties of a calcite (10.4) surface. Our results show that ethanol and/or pentanol molecules form a well-ordered monolayer (through their hydroxyl group with carbon chains sticking away in a standing-up position) on the calcite (10.4) surface. Additionally, we found intriguing modulations in the photoabsorption spectra and circular dichroism spectra. In particular, the latter was a unique optical fingerprint for a molecule-adsorbed calcite (10.4) surface. Our findings provide useful insights into the structural and optical features of calcite-based systems at the atomic level.

## 1. Introduction

Molecular adsorption on mineral surfaces has been a prominent research area in the field of surface chemistry. Among minerals, calcium carbonate (Ca${}^{2+}$CO${}_{3}^{2-}$) has attracted significant attention because it is the most abundant mineral in nature, constituting 4% by weight of the Earth’s crust [1,2,3,4,5,6]. Calcium carbonate has three polymorphs: calcite, aragonite, and vaterite. Among them, calcite is thermodynamically the most stable polymorph. It is the main constituent of sedimentary rocks such as chalk and limestone, where a large percentage of the world’s oil reserves are found. It is also an important component in bones, teeth, shells of marine organisms and biomineralization [7,8], and has promising applications in pharmaceuticals, paint, pigments, optical devices and a wide range of other products [9,10]. Calcite surfaces are hydrophilic, but a single monolayer of organic molecules can change their behavior from hydrophilic to hydrophobic [11]. Reports of the stability of various calcite surfaces and their molecular adsorption are widely available [12,13,14,15,16,17,18,19,20,21,22,23,24,25,26,27,28,29,30,31]. Notably, a previous work combining X-ray photo-electron spectroscopy and molecular dynamics simulations showed that alcohols interact with the calcite surface through the OH group, with their C chains sticking away in a standing-up orientation. In addition, it was reported that the length of the alcohol molecule does not influence the surface coverage or structure of the monolayer but the coverage decreases when the alcohol is bulky [20]. However, in spite of these previous works, it is still an intriguing question as to how exactly at the atomic level, molecular ordering and molecular length affect calcite optical properties. Our computational study aimed to fill this gap.

**Figure 1:**
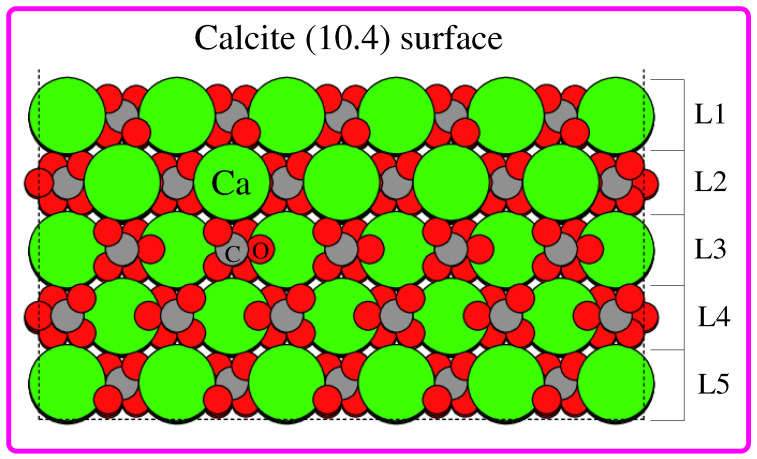
Pristine calcite (10.4) surface of 8 × 8 dimension consisting of 5 layers, namely L1, L2, L3, L4 and L5. Green, gray and red spheres depict Ca, C and O atoms, respectively.

In the present study, using first-principle quantum mechanical calculations, we aimed to establish some useful insights into the role of molecular ordering and molecular length on calcite optical properties. We chose the calcite (10.4) surface: the most stable plane of calcite which dominates the observed morphology [32,33,34], a structural model is presented in Figure 1. In short, calcite (10.4) surface contains both Ca${}^{2+}$ and CO${}_{3}^{2-}$ ions held together via ionic bonding, making its charge neutral and having a zero dipole moment [35]. Calcite (10.4) surface has a higher density of Ca${}^{2+}$ and CO${}_{3}^{2-}$ ions compared to other possible neutral planes, leading to its stability with a surface energy of 0.59 J m${}^{-2}$ [16]. In a related context, the list of organic molecules that one could select is substantial. Nevertheless, we chose ethanol (C${}_{2}$H${}_{6}$O) and pentanol (C${}_{2}$H${}_{12}$O). Note that in common, they have a CH${}_{3}$ end, and a polar, OH end. To find the most stable adsorption geometry for a particular organic molecule, calculations were performed with a number of starting geometries through an exhaustive sampling of all possible adsorption sites with full monolayer coverage.

The remainder of this paper is organized as follows. In the following section, we provide the details of the computational methodology we used. Then, we discuss the key results from our simulations such as the relative stability, electron difference densities, photoabsorption spectra and circular dichroism spectra. Our results show that ethanol and/or pentanol molecules form a well-ordered monolayer (through the OH group with their C chains sticking away in a standing-up orientation) on the calcite (10.4) surface in agreement with experiment findings and previous calculations. We also find intriguing modulations in the photoabsorption spectra and circular dichroism spectra of a molecule-adsorbed calcite (10.4) surface. The calculated results will provide fundamental insights into the role of molecular ordering and molecular length on the optical properties of the calcite (10.4) surface.

## 2. Computational Methodology

Ground state density functional theory (DFT) calculations were carried out using QuantumWise Atomistix ToolKit (QuantumATK) software [36] with the numerical LCAO (linear combination of atomic orbitals) basis sets [37] and PBE/GGA [38] exchange and correlation functional. London dispersion corrections were included through the semiempirical DFT-D3 method [39] of Grimme with modified C${}_{6}$ parameters for ionic solids [40,41,42]. Note that the important role played by dispersion corrections on the calcite surface has been reported by a number of previous DFT calculations [42,43]. In the case of structure optimizations, the electronic energy, total energy and force convergence thresholds were set to 10${}^{-8}$, 10${}^{-4}$ and 10${}^{-3}$ in atomic Rydberg units, respectively. We modeled the calcite (10.4) structure with 8 × 8 dimensions consisting of five layers (L1, L2, L3, L4 and L5; see Figure 1), to which 30 Å of vacuum was added at the top (perpendicular to the surface). The calcite (10.4) structure we modeled is composed of 693 atoms: 117 Ca, 114 C and 346 O. One of our initial calculations revealed that when all atoms of the calcite (10.4) structure are allowed to relax, the surface structure has been considerably distorted at the edges. Therefore, a few geometric constraints were introduced for the structure optimizations. During the structure optimization of the pristine calcite (10.4) surface, we allowed the atoms in the top two layers (L1 and L2 in Figure 1) to relax while the atoms in the three bottom layers (L3, L4 and L5 in Figure 1) were held fixed. Similarly, in the case of hybrid systems (molecule-adsorbed calcite (10.4) surface), we allowed both the molecules and top two layer atoms of calcite (10.4) to relax while fixing the three bottom layers of calcite (10.4). These geometrical constraints retained the 2D symmetry of the calcite (10.4) surface while significantly reducing the edge effects. Note that a full monolayer coverage of molecules on the calcite (10.4) surface requires 15 ethanol and/or pentanol molecules.

The photoabsorption spectra and circular dichroism spectra in this work were calculated using a highly efficient sTDA method (simplified TDA (Tamm–Dancoff approximation)) by Grimme [44,45,46,47,48,49]. We employed the sTDA method instead of the full-time dependent density functional theory (TD-DFT) method due to the large size of investigated systems. We used the double-ζ polarized (dzp) basis sets and PBE exchange and correlation functional. More accurate hybrid functionals such as PBE0 [50,51] and HSE [52,53] would improve the results but are infeasible to use in this work because of the very large size of the investigating systems. Note that a full TD-DFT calculation (in a typical photoabsorption range from 0 to 7 eV) is feasible for systems with up to approximately 200–300 atoms. However, full TD-DFT calculations with several hundreds of atoms are extremely time-consuming since one needs to solve the following non-Hermitian eigenvalue problem:

$$\left(\begin{array}{cc} A & B\\ B^{*} & A^{*} \end{array}\right)\left(\begin{array}{c} X\\ Y \end{array}\right)=\left(\begin{array}{cc} \omega & 0\\ 0 & -\omega \end{array}\right)\left(\begin{array}{c} X\\ Y \end{array}\right)$$

where A and B are the so-called orbital rotation Hessian matrices with eigenfunctions X and Y and ω is a vector with the dimension of the number of roots that contains the respective eigenvalues. Furthermore, elements of matrices A and B take the form; $A_{ia,jb}=\delta_{ij}\delta_{ab}\left(\epsilon_{a}-\epsilon_{i}\right)+2\left(ia|jb\right)-a_{x}\left(ij|ab\right)+\left(1-a_{x}\right)\left(ia\left|f_{Xc}\right|jb\right.$ and $B_{ia,jb}=2\left(ia|bj\right)-a_{x}\left(ib|aj\right)+\left(1-a_{x}\right)\left(ia\left|f_{XC}\right|bj\right).$ Here, $\epsilon_{a}$ and $\epsilon_{i}$ are the orbital energies of the virtual and occupied orbitals obtained from the respective DFT ground state calculation. In the TDA method, the matrix **B** is neglected. Further three simplifications produce the sTDA method: (i) neglecting the response of the exchange-correlation functional; (ii) evaluating the two-electron integrals as damped Coulomb interactions between transition/charge density monopoles; and (iii) restricting the configuration space to a user-specified energy range of excitations. We used the default configuration selection energy threshold of 10${}^{-4}$. Briefly, sTDA is currently the method of choice for calculating the optical properties of large systems up to 1000 atoms. sTDA makes use of simplifications in the treatment of molecular two-electron integrals and massively truncates the single excitation expansion space. This leads to computational savings compared to full TD-DFT by at least two orders of magnitude at only a minor loss of accuracy in typical applications. The sTDA excitation energies are of good quality with typical deviations of 0.2–0.3 eV from the experimental data [45,54].

## 3. Results and Discussion

Before analyzing/discussing the structural, energetic and optical properties of the molecule-adsorbed calcite (10.4) surface, it is useful to begin our discussion, starting from the geometric features of the bulk calcite and calcite (10.4) surface. Briefly, calcite consists of positively charged Ca${}^{2+}$ and negatively charged CO${}_{3}^{2-}$. In the bulk structure, each Ca atom is bonded to six O atoms (an experimentally measured Ca–O bond distance of 2.36 Å) and each O atom is bonded to one C atom and two Ca atoms. Now, regarding the calcite (10.4) surface, it is formed by the rupture of the ionic bonds with Ca and the top surface O atoms which are under coordinated compared with the bulk, as can be seen in the calcite (10.4) surface in Figure 1. The numbers of under coordinated Ca and O atoms are equal, so the charge of the calcite (10.4) surface is neutral. The topmost O atoms are under coordinated and their high partial negative charge makes them good hydrogen bond acceptors. Additionally, Ca is under coordinated so its partial positive charge makes it an anchor point for negatively charged species. In the present study, the initial geometries for ethanol and/or a pentanol-adsorbed calcite (10.4) surface was generated by placing the molecules at different positions with a full monolayer coverage. Note that a full monolayer coverage corresponds to 15 molecules. We identified eight unique full monolayer configurations for each calcite(10.4)/ethanol and calcite(10.4)/pentanol system. More specifically, we sampled one on-top (a surface site that is 1-fold coordinated) O site, one on-top C site, five bridge (a surface site that is 2-fold coordinated) sites and one HCP (a 3-fold coordinated surface site that corresponds to hexagonal-close-packed stacking) site. The molecules were placed at a height of 2.50 Å above the absorption sites. The calcite (10.4) surface and adsorbed molecules were then allowed to relax to their minimum energy configuration subjected to the geometrical constraints, as mentioned previously.

**Figure 2:**
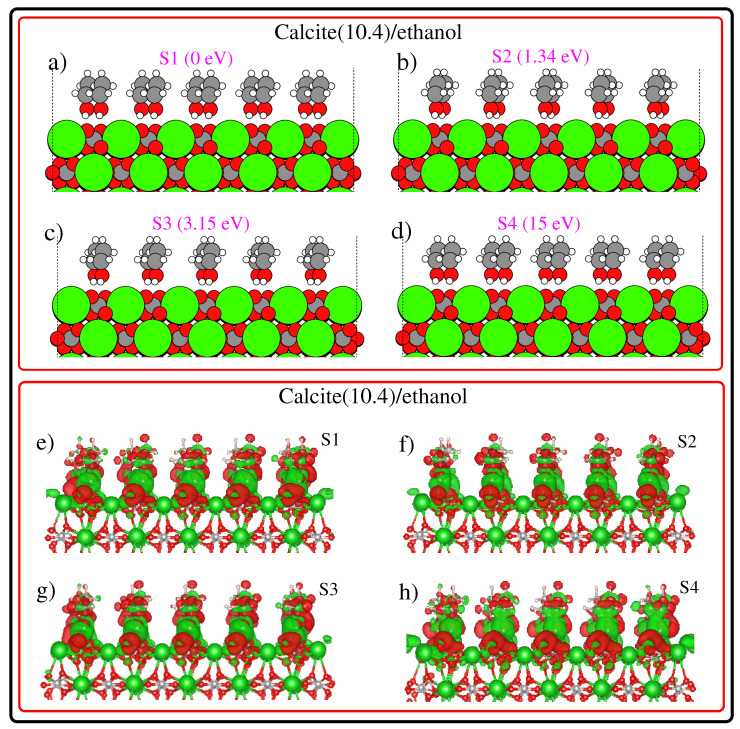
The first four low-energy calcite(10.4)/ethanol configurations (**a**–**d**) obtained from our calculations. The most stable configuration is labeled S1 and the least stable configuration is labeled S4. The relative stability is given in the inset. Electron difference density plots of calcite(10.4)/ethanol configurations (**e**–**h**). The green/red region depicts accumulation/depletion in electron density. EDDs are plotted using a density isovalue of 0.0005 electrons/Bohr${}^{3}$.

The first four low-energy configurations of calcite(10.4)/ethanol systems, along with their relative stability, are summarized in subplots (a), (b), (c), (d) of Figure 2. Among these low energy configurations, S1 (S4) is the most (least) stable. Upon inspecting the calcite(10.4)/ethanol-optimized configurations, we found an inward relaxation of the topmost L1 surface. This inward relaxation is a direct consequence of surface asymmetry, where the force of attraction will be greater for bulk atoms causing a significant inward relaxation of the top surface layer. In addition, as a common effect which appears in all configurations shown in Figure 2, the surface CO${}_{3}^{2-}$ groups are tilted from their ideal positions. The ordering of ethanol molecules suggests that they attach as a brush protruding out from the calcite (10.4) surface. Ethanol, with its CH${}_{3}$ end and its polar OH end, is forced by chemical affinity to orient with the OH end toward the ionic calcite (10.4) surface. The fatty CH${}_{3}$ ends of the molecules are oriented away from the calcite (10.4) surface, as can be seen in Figure 2. This result is in good agreement with experimental findings [55]. Now let us further analyze the structural features of the minimum energy configurations shown in Figure 2 starting from the most stable S1 configuration (relative stability of 0 eV). Note that, in the S1 structure, the ethanol molecules occupy the calcite (10.4) bridge sites (not shown in Figure 2). The second low energy structure S2 (ethanol molecules positioned on-top C sites) is 1.34 eV less stable compared than the S1 structure. On inspecting the ethanol arrangement in S1 and S2 from Figure 2, the differences in molecular ordering are apparent. The next low energy configuration S3 is 3.15 eV higher in energy than the S1 structure and ethanol molecules in S3 are located on the bridge sites. One important observation from Figure 2 is that ethanol molecules form a well-ordered structure more or less perpendicular to the calcite (10.4) surface, with their OH group in close proximity with the surface and C chains sticking away in a standing-up position, in agreement with the experimental results. In this context, it worth mentioning that Haddad and co-workers [56] reports that the thickness of the alcohol monolayer becomes slightly thinner when raising the temperature above the alcohol melting point, indicating that all molecules are more or less aligned with the normal surface. Then, we compare the electron difference density (EDD) plots of S1, S2, S3, and S4, see subplots (e), (f), (g), (h) of Figure 2. More specifically, EDD in this work was calculated by taking the difference between the electron density of the full system minus the electron density of the surface without the adsorbates, minus the electron density of the adsorbates without the surface. Remarkably, the EDD renders valuable information about the formation of bonds and charge redistribution between the subsystems. Apparently, in common to all EDD plots, H atoms of ethanol molecules are electron deficient. This observation is in tune with the X-ray reflectivity experiments. Note that X-ray reflectivity experiments probe the electron density profile at interfaces, which is suitable for studying thin layers on flat substrates [57,58,59,60].

**Figure 3:**
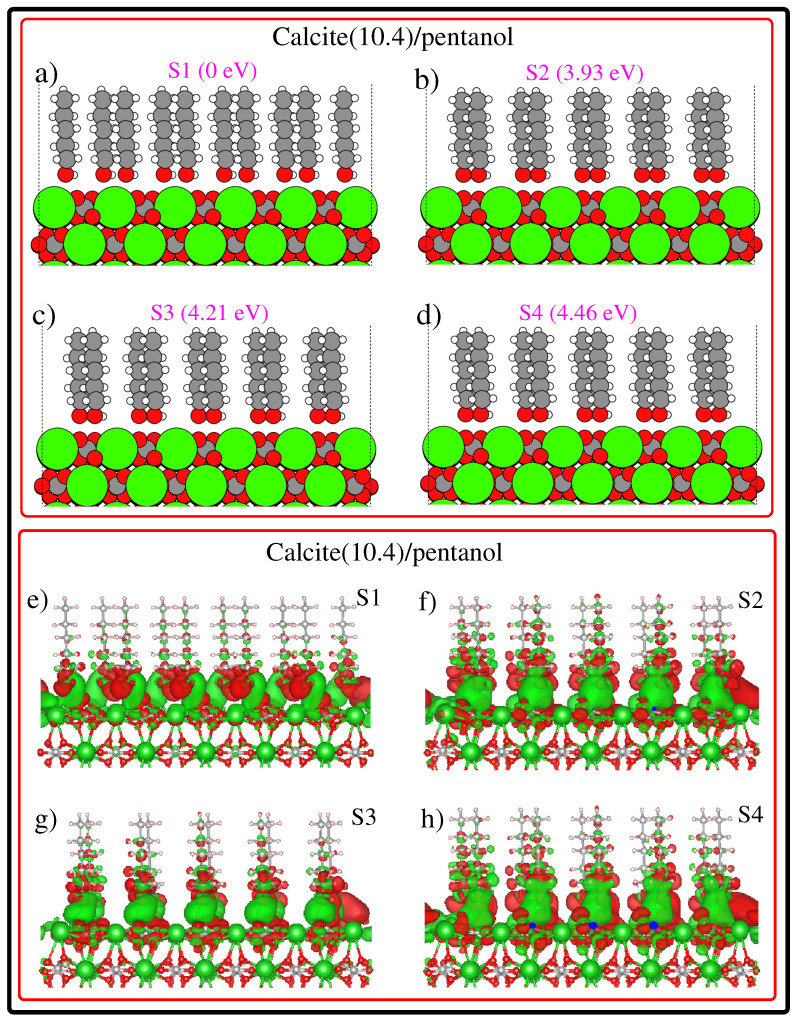
The first four low-energy calcite(10.4)/pentanol configurations (**a**–**d**) emerged from our calculations. The most stable configuration is labeled by S1 and the least stable configuration is labeled by S4. The relative stability is given in the inset. Electron difference density plots of calcite(10.4)/pentanol configurations (**e**–**h**). The green/red region depicts an accumulation/depletion in electron density. EDDs are plotted using a density isovalue of 0.0005 electrons/Bohr${}^{3}$.

After having discussed the first four stable configurations and their corresponding EDD plots for the calcite (10.4)/ethanol configuration, we then discussed the stable configurations and their corresponding EDD plots for calcite(10.4)/pentanol configuration, as can be seen in Figure 3. One may easily conclude that pentanol molecules present a well-ordered monolayer on top of the calcite (10.4) surface. Pentanol, with its CH${}_{3}$ end and its polar OH end, is forced by chemical affinity to orient with the OH end toward the ionic calcite (10.4) surface. This finding is in agreement with the experimental results [55] and previous theoretical calculations by Bovet and co-workers [20]. Now, let us briefly discuss the energetic ordering of calcite(10.4)/pentanol configurations. Figure 3 subplots (a), (b), (c) and (d) shows that S2, S3 and S4 configurations are 3.93 eV, 4.21 eV and 4.46 eV less stable than the S1 structure, respectively. In the case of the S1 structure, the pentanol molecules are positioned over the bridge sites (not shown in Figure 3) of the calcite (10.4) surface. This result shows that despite the changes in molecular length, both ethanol and pentanol molecules favor bonding with the calcite (10.4) surface via bridge sites. This is because the bridge sites enhance bonding via multiple bonds. Additionally, the inspection of EDD plots in Figure 3 reveals that H atoms of pentanol molecules are highly electron deficient. The strong affinity of molecules to bind on a calcite (10.4) surface is consistent with the available results [61].

**Figure 4:**
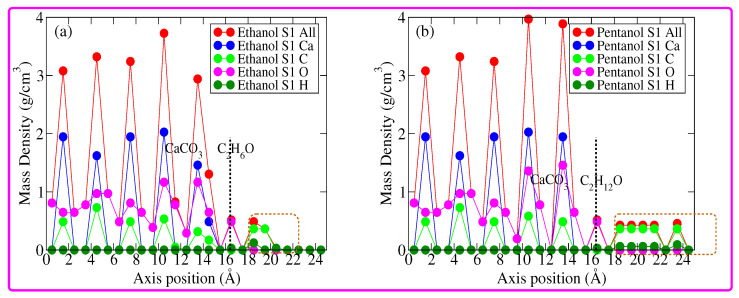
(**a**) Mass density profiles of the calcite(10.4)/ethanol S1 configuration; and (**b**) same as before, but for calcite(10.4)/pentanol S1 configuration.

Further details regarding the molecular ordering on top of the calcite (10.4) surface can be obtained by analyzing the mass density profiles (MDP) of the most stable configurations, see Figure 4. Note that in Figure 4, subplot (a) corresponds to the MDP of the calcite(10.4)/ethanol S1 configuration and subplot (b) corresponds to the MDP of the calcite(10.4)/pentanol S1 configuration. Figure 4 shows that the calcite (10.4) surface MDP lies in the range of 1–14.5 Å (see the vertical dashed line). Above this range, the MDP of adsorbed molecules becomes apparent. The MDP of the calcite(10.4)/pentanol S1 configuration is more extended in space (in the range of 1–24 Å) than the MDP of the calcite(10.4)/ethanol S1 configuration (in the range of 1–20 Å). This result is not surprising since the pentanol molecule is more or less double in length that of the ethanol molecule. Let us now discuss the MDPs in detail. An MDP of calcite(10.4)/ethanol S1 configuration reveals that at approximately 2.0 Å above the calcite (10.4) surface, the first peak (shown in a dashed rectangular area) is mostly contributed by O atoms. While the second and third peaks are mostly contributed by C atoms. This result confirms the high degree of ordering and the upright configuration of ethanol molecules on the calcite surface (10.4). Interestingly, the MDP of the calcite(10.4)/pentanol S1 configuration exhibits several peaks. Apparently, the first peak above the calcite surface is largely contributed by O atoms. This indicates that pentanol molecules attach through the OH end. Inspecting the rest of the peaks shows a high degree of ordering and the upright configuration of pentanol molecules on the calcite (10.4) surface.

**Figure 5:**
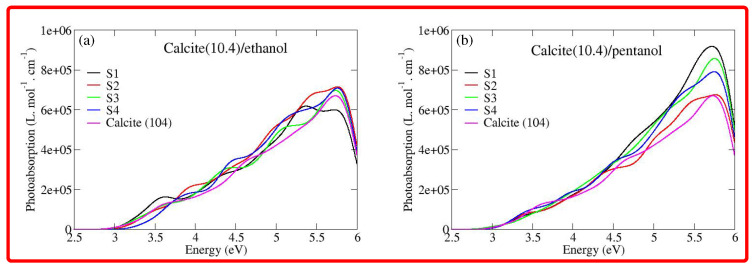
(**a**) sTDA calculated the photoabsorption spectra of calcite(10.4)/ethanol configurations; and (**b**) the same as before but for calcite(10.4)/pentanol configurations. In each plot, we also provided the photoabsorption spectrum of the pristine calcite (10.4) surface. 2e+05 represents 2 × 10${}^{5}$, the same rule applies to other E notations.

We then analyze/compare the modulations in the photoabsorption spectra of the calcite(10.4)/ethanol configurations and calcite(10.4)/pentanol configurations, as can be seen in Figure 5. To make the comparison useful, we also present the photoabsorption spectrum of the pristine calcite (10.4) surface. Let us begin our discussion by starting with the photoabsorption spectra of calcite(10.4)/ethanol configurations, as can be seen in subplot (a) of Figure 5. As expected, the photoabsorption spectra of the calcite(10.4)/ethanol configurations are distinct in comparison with the pristine calcite (10.4) surface. In addition, the photoabsorption spectra of calcite(10.4)/ethanol configurations are also more or less distinct from each other. This is not surprising, since the different ordering of ethanol molecules on top of the calcite (10.4) surface would instigate different amounts of interfacial charge transfers (as evidenced earlier from EDD plots in Figure 2) and consequentially affects the photoabsorption process. Figure 5 shows that, in the case of a pristine calcite (10.4) surface, the photoabsorption spectrum is characterized by three peaks, centered at approximately 3.60 eV, 4.60 eV and 5.75 eV, respectively. The calcite(10.4)/ethanol S1 configuration produces clear modulations in the photoabsorption spectra. In particular, the intensity of the first peak is somewhat enhanced in comparison with the pristine calcite (10.4) case. The influence of different adsorption sites on the photoabsorption spectra is apparent throughout the whole absorption range since different absorption sites would yield different charge transfers between the calcite (10.4) and ethanol molecules. Now, let us briefly discuss the modulations in the photoabsorption spectra of calcite(10.4)/pentanol configurations, as can be seen in subplot (b) of Figure 5. Apparently, in contrast to the photoabsorption spectra of calcite (10.4)/ethanol configurations, one finds that the photoabsorption spectra of calcite(10.4)/pentanol configurations are clearly distinct from the photoabsorption spectrum of the pristine calcite (10.4) surface. This can be attributed to the increased chain length of the pentanol in comparison to the ethanol molecule. In general, although the photoabsorption spectra of calcite(10.4)/ethanol configurations and calcite (10.4)/pentanol configurations show some differences, the different systems cannot be completely discriminated just by looking at the photoabsorption spectra. In parallel efforts, electronic circular dichroism (ECD) [62,63,64,65,66,67,68] spectra would help discriminate the different systems since it is highly sensitive to small details in the atomic structure and unique for each configuration [69].

**Figure 6:**
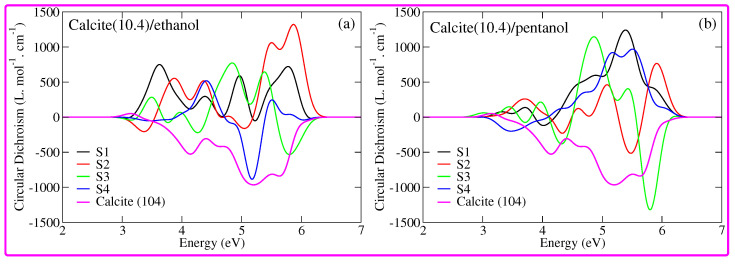
(**a**) sTDA calculated circular dichroism spectra for calcite(10.4)/ethanol configurations; and (**b**) the same as before but for calcite(10.4)/pentanol configurations. In each figure, we also provided the circular dichroism spectrum of the pristine calcite (10.4) surface.

A commonly used experimental quantity to measure ECD spectra is the difference in molar extinction coefficient: $\Delta \epsilon \left(\omega \right)=\frac{16\pi N_{A}}{3\ln\left(10\right)10^{3}}\frac{2\pi }{\hbar c}\omega R{\left(\omega \right)}^{\mathrm{cgs}}$. Here, ω is the energy of the incident light, *c* is the speed of light, *ℏ* is the reduced Planck constant, $N_{A}$ is Avogadro’s constant, and $R{\left(\omega \right)}^{\mathrm{cgs}}$ is the rotatory strength in cgs units. The quantity that characterizes Δϵ(ω) and therefore the EDS spectrum is the rotatory strength. ECD spectra of calcite(10.4)/ethanol configurations and calcite(10.4)/pentanol configurations are shown in Figure 6a,b, respectively. First, we start with the calcite(10.4)/ethanol configurations, as can be seen in the subplot (a) of Figure 6. In the case of a pristine calcite (10.4) structure, the ECD spectrum is composed of several bands. The first band is a weak positive peak centered at approximately 3.10 eV and all other peaks are strong negative with a maximum negative peak intensity recorded at 5.15 eV. This scenario changes in the case of the calcite(10.4)/ethanol S1 configuration. One finds that its ECD spectrum is composed of four strong positive peaks with strong peaks located at the start and at the end of the spectrum. The ECD spectrum of calcite(10.4)/ethanol S2 configuration also exhibits strong positive peaks. Furthermore, S3 and S4 configurations exhibit strong positive and negative peaks. We remark that the differences in the ECD signals of the calcite(10.4)/ethanol configurations can be attributed to the different ordering of ethanol molecules. Apparently, ECD spectra reveal that it is an ideal technique to optically discriminate between different systems. Finally, one finds that calcite(10.4)/pentanol configurations can also be clearly discriminated through their ECD spectra. Overall, the ECD spectra provide the optical fingerprint to discriminate between the molecule-adsorbed calcite (10.4) configurations.

## 4. Conclusions

In this computational study, we reported the impact of the adsorption of straight chain alcohol (ethanol and pentanol) molecules on the structural, energetic, and optical properties of the calcite (10.4) surface. Ethanol and pentanol molecules bind to the calcite (10.4) surface via their OH functional group, creating a well-ordered monolayer on top of the calcite (10.4) surface. We find intriguing modulations in the photoabsorption spectra and electronic circular dichroism spectra of the molecule-adsorbed calcite (10.4) surface. More interestingly, an electronic circular dichroism spectra rendered the optical fingerprint to discriminate between different calcite (10.4)/ethanol and calcite(10.4)/pentanol configurations. Our theoretical findings reveal several unique aspects of the optical properties of molecule-adsorbed calcite (10.4) surfaces. Understanding how simple straight chain alcohol molecules modify the optical properties of a calcite surface is a step toward understanding biomineralization processes in more detail.

## Data Availability

Not applicable.
